# Virotherapy as a Potential Therapeutic Approach for the Treatment of Aggressive Thyroid Cancer

**DOI:** 10.3390/cancers11101532

**Published:** 2019-10-10

**Authors:** Anna Maria Malfitano, Sarah Di Somma, Nella Prevete, Giuseppe Portella

**Affiliations:** Dipartimento di Scienze Mediche Traslazionali, Università Federico II Napoli, URT “Genomic of Diabetes” of Institute of Experimental Endocrinology and Oncology “G. Salvatore”, National Council of Research (CNR), 80131 Naples, Italy; annamaria.malfitano@unina.it (A.M.M.); nella.prevete@unina.it (N.P.)

**Keywords:** oncolytic virus, immunotherapy, anaplastic and poorly differentiated thyroid carcinoma

## Abstract

Virotherapy is a novel cancer treatment based on oncolytic viruses (OVs), which selectively infect and lyse cancer cells, without harming normal cells or tissues. Several viruses, either naturally occurring or developed through genetic engineering, are currently under investigation in clinical studies. Emerging reports suggesting the immune-stimulatory property of OVs against tumor cells further support the clinical use of OVs for the treatment of lesions lacking effective therapies. Poorly differentiated thyroid carcinoma (PDTC) and anaplastic thyroid carcinoma (ATC), have a poor prognosis and limited treatment options. Therefore, several groups investigated the therapeutic potential of OVs in PDTC/ATC models producing experimental data sustaining the potential clinical efficacy of OVs in these cancer models. Moreover, the presence of an immunosuppressive microenvironment further supports the potential use of OVs in ATC. In this review, we present the results of the studies evaluating the efficacy of OVs alone or in combination with other treatment options. In particular, their potential therapeutic combination with multiple kinases inhibitors (MKIs) or immune checkpoint inhibitors are discussed.

## 1. Introduction

Thyroid carcinoma (TC) is the most common endocrine malignancy. Its incidence is estimated to be 1–2 million per year [1,2]. Papillary thyroid carcinoma (PTC) comprises 80–85% of all thyroid neoplasms, follicular thyroid carcinoma (FTC) accounts for 10–15% of cases, and PDTC and ATC are the less common histotypes. PTC and FTC have an excellent survival rate (98%) and a good prognosis, with a low recurrence rate (5%), whereas PDTC and ATC have a poor prognosis and account for most of the TC related deaths. ATC and PDTC are tumors that lose, completely or partially, the typical features of their original follicular cells. Genetic analysis of these tumors demonstrated that ATC, and to a lesser extent PDTC, accumulate several different oncogenic alterations, TERT oncogene and TP53 tumor suppressor gene are the most frequently altered genes. Other pathways like MAPK and PI3K-AKT are altered in these tumors with mutation in BRAF, RAS, AKT, PTEN, and PIK3CA [3]. Undifferentiated TCs are characterized by a rapid tumor growth, trachea obstruction with respiratory distress, and frequent distant metastases, with a survival time of six months from the diagnosis.

For these forms multimodal treatment, surgery, chemotherapy, and radiation obtained a very limited success [1,2,4,5]. Multiple kinase inhibitors (MKIs) targeting the signaling pathways oncogenically activated in TC (i.e., RAS, BRAF, MEK, MAPK) are currently used to treat the advanced forms, exerting cytostatic effects on TC cells. However, the development of tumor resistance to these MKIs has limited their clinical benefits [6,7] and not increased the survival rate for ATC or PDTC.

The need for more effective therapies has prompted the investigation toward different approaches including virotherapy with OVs that selectively infect and lyse cancer cells, without harming normal cells or tissues [8,9]. Studies performed in experimental models of ATC sustain the potential clinical efficacy of OVs in the treatment of ATC/PDTC in combination with different agents. In this review, we focused on reports dealing with the use of OVs in TC.

## 2. Virotherapy and OVs

The hypothesis that cancer cells are vulnerable to viral infections is very old. In early 1900s, the observation of an Italian gynecologist, Dr. Di Pace and several others, reported cancer remission after viral infections [10]. Thus, virotherapy was hypothesized to be useful against cancer and some clinical trials were performed between 1950 and 1970 [11]. However, severe side effects and treatment related deaths were observed, thus the use of viruses for cancer treatment was considered unsafe.

In 1990s, the research on OVs revived owing to a better knowledge of viral gene functions, a better understanding of the molecular alterations in cancer cells, and the availability of technologies for the manipulation of viral genomes. Two studies had a crucial role for the development of this research area:

i) In 1991, a modified Herpes Simplex Virus 1 (HSV-1) with reduced neurotoxicity and with a mutation in the thymidine kinase gene was shown to replicate selectively in cancer cells [12];
ii) In 1996, *dl*1520 (a.k.a. ONYX-015), an oncolytic adenovirus (Ads) with the deletion of *E1B55K* gene was described. E1B55K product binds and degrades p53. In most neoplastic cells, the functional inactivation of p53 complements *E1B55K*-deletion or viral mRNA nuclear export [13,14]. *dl*1520 has been evaluated in 18 phase I and II clinical trials confirming its safety [15], however a phase III clinical trial has not been completed because of changes in the priority choice of the company [16].

Since then, different strategies to obtain tumor selectivity or to increase the efficacy of OVs have been exploited, and different viral strains tested for tumor selectivity. An increasing number of viruses are under investigation in clinical studies [17]; three viruses are currently approved for cancer treatment.

Rigvir, a wildtype non-pathogenic enteric cytopathic human orphan type 7 (ECHO-7) was approved only by the Latvian regulatory agency for the treatment of melanoma [18,19]. This virus has not met the required standard regulation for European or US approval, further studies are in progress to better evaluate its therapeutic potential [20].

In 2005, H101, an Ads with the same deletion of *dl*1520, was approved by China’s State Food and Drug administration for the treatment of head and neck carcinoma on the basis of the results obtained in a Phase III trial [21,22].

In 2015, EMA and FDA approved T-VEC (talimogene laherparepvec, Amgen, Inc., Thousand Oaks, CA, USA), a modified HSV-1 expressing GM-CSF [23]. T-VEC is now included as a routine treatment option for unresectable early metastatic melanoma, but it has also been used as neo-adjuvant therapy and in other cancer types [24].

Currently, other OVs are being tested in clinical trials (https://clinicaltrials.gov/ClinicalTrials.gov:NCT002705;NCT0320601, NCT03004183; etc.) and although it is likely that not all of them will be effective, the number of OVs approved for cancer treatment is expected to increase in future.

## 3. Direct Effects of OVs

The tumor selectivity of OVs can be either natural or obtained by viral genome manipulation. On this basis, OVs are divided in two groups: natural or genetically modified.

The natural OVs have innate selectivity toward cancer cells, exploiting the aberrant signaling pathways that sustain cancer growth [25].

Genetically modified OVs are viruses whose genome has been manipulated, to selectively replicate in neoplastic cells. To obtain a selective viral infection/replication in tumor cells, the most common strategies focused on restricting the expression of essential viral-proteins by using cancer-specific promoters, or deleting viral proteins that prevent apoptosis in healthy cells [25,26]. Other approaches include the selective control of replication via viral gene inactivation, transcriptional targeting, microRNA targeting sequences [17].

Other modifications include the insertion of therapeutic genes in order to generate “armed” OVs with the aim to improve therapeutic efficiency and selectivity. Some modifications were made to improve the direct cytotoxic effects of viruses on cancer cells. This class of modifications includes the insertion in the viral genome of therapeutic transgenes to increase release, spread, and to reduce off-target effects on normal cells. Other viruses have modification of the capsid to improve the therapeutic window. The last generation of armed OVs combines OVs with immune modulators in order to enhance the efficiency of immune response and of OVs. These last OVs are engineered to express, cytokines or chemokines [27]. Among several others (i.e., reporter genes, radio-sensitizers, prodrug convertases), OVs have been engineered also to express anti-angiogenic genes as transgenes to target tumor vasculature [28]. Cytokines and chemokines can stimulate simultaneously different immune cells favoring immune response against cancer cells. Several OVs expressing these molecules have been generated.

The capability of OVs to selectively replicate in and lyse cancer cells sparing normal cells not only relies on the selective replicative activity (natural or obtained) of the virus, but also on the reduced anti-viral response of neoplastic cells. Indeed, in normal cells viral infection elicits the release of interferons (IFNs), produced following the detection of viral RNA, DNA, or proteins by intracellular pathogen recognition receptors (PRRs). IFN signaling pathway activates the expression of effector genes known as interferon-stimulated genes (ISGs). The expression of ISGs aims to the clearance of the viral infection [28,29]. Neoplastic cells have abnormal IFN pathways. The loss of IFN type I signaling allows cancer cells to circumvent the growth inhibitory effects mediated by IFN [30,31] and mediates immune evasion of tumor [32]. IFN pathway abnormalities are more frequent in advanced aggressive lesions and therefore OVs might display a higher proliferative potential in more aggressive cancers [33]. Overall, IFN pathway abnormalities favor cancer cell survival and immune-escape, but make neoplastic cells highly susceptible to viral infection.

Other alterations of anti-viral response also contribute to OV selectivity for neoplastic cells. Protein kinase R (PKR) pathway [34] is critical for intrinsic cellular anti-viral response. The recognition of viral elements by Toll-like receptor (TLR) activates the downstream pathways, leading to the activation of PKR [35]. In normal cells, phosphorylated PKR inhibits protein synthesis with the consequent block of viral replication and propagation [36]. To support uncontrolled proliferation in neoplastic cells, the PKR pathway is not functional, thus allowing viral replication. Mutations of RAF or NRAS, frequently observed in TC, constitutively activate MAP kinase pathway, which suppresses PKR activation [37,38]. In TCs, a reverse correlation between PKR and Ki-67 expression has been observed, indicating that TC cells might be susceptible to OV infections [39].

## 4. Indirect Effects of OVs

Indirect effects of OVs are mainly associated with the capability of OVs to stimulate an anti-tumor immune response, therefore, OVs are also considered as immunotherapeutic agents. The main indirect effects of OVs are listed below [40], and reported more in detail in the next paragraphs:

i) Activation of danger signals and immunogenic cell death (ICD) pathways with the subsequent stimulation of innate or adaptive immune responses against cancer cells.
ii) Release of antigens derived from both the virus and cancer cells, leading to immune activation also against non-infected tumor cells.
iii) Release by infected dying cells of several cytokines into the local environment (i.e., IFNs, tumor necrosis factor-alpha and interleukins) that further promote immune response and reshape the pro-tumor microenvironment (TME) toward an inflammatory and anti-tumor phenotype.
iv) Inhibition of tumor angiogenesis because of the interaction of OVs with TME.

### 4.1. Activation of ICD Pathways by OVs

Cell death pathways activated by OV infection are highly immunogenic [41,42] and include pyroptosis (caspase-1-dependent cell death), autophagic cell death, ICD, necrosis, necroptosis, and apoptosis, although the latter is essentially immunoquiescent. ICD is defined as a form of regulated cell death sufficient to activate an adaptive immune response in an immunocompetent host [43]. ICD results in the release of danger associated molecular patterns (DAMPs) initiating immune response. Three DAMPs are considered as biochemical hallmarks of ICD: calreticulin, HMGB1, and ATP. Calreticulin (CRT) exposed on the surface, or released in the surrounding milieu, of a dying cell acts as a phagocytic signal to trigger an innate immune response [44,45]. HMGB1 triggers an inflammatory response via interaction with specific receptors [46]. The extracellular release of ATP acts as a potent chemoattractant of immune cells.

Antigen presenting cells (APCs), such as dendritic cells (DCs) and macrophages, in TME recognize the danger signals of ICD, generating an immune response and the production of alpha and beta interferons. Cytokines released by activated DCs contribute to the activation of natural killer (NK) cells [47]. Furthermore, macrophages conditioned toward an M1-phenotype by IFN signals, directly contribute to tumor cell killing.

The ability of OVs to sustain ICD has been associated to their capability to release DAMPs or soluble tumor-associated antigens that in turn induce innate and adaptive immune responses [48]. The activation of ICD has been reported for OVs of different origin [49,50,51]. Indeed, in culture Ads [52], measles virus [53], oncolytic reovirus [54], and coxsackievirus B3 [55] were shown to induce all the key features of ICD (i.e., surface expression of calreticulin, higher levels of extracellular ATP and HMGB1) in several cancer models [56]. These evidences were also confirmed in vivo in immunocompetent mice models demonstrating the ability of Ads to increase the infiltration, in ectopic tumor transplantations, of macrophages and T cells with an anti-tumor/cytotoxic phenotype [52]. Although defective of adaptive immune system also in nude mice, coxsackievirus B3 OV displayed the ability to recruit an increased number of innate immune cells in xenotransplants [55].

Several studies both in preclinical models and in human patients approached the possibility to improve the immunogenicity of cancer cells by the combination of OVs with drugs able to initiate ICD. hTert-Ads (human telomerase reverse transcriptase promoter-regulated Ads) in combination with the proteasome inhibitor bortezomib enhanced endoplasmic reticulum (ER) stress and ICD thus sustaining in vivo anti-tumor activity in hepatocellular carcinoma [57]. Similarly, a preclinical murine breast cancer model was used to verify that combination of oncolytic HSV-1 with mitoxantrone, a drug known to induce ICD, enhanced therapeutic efficacy of the drug by abolishing tumor immune tolerance [58]. In chemotherapy refractory pancreatic carcinoma patients, the administration of Ads in combination with temozolomide and cyclophosphamide prolonged the disease control and survival. The increased survival was significantly associated to an increase in serum HMGB1 levels in turn associated to an increased anti-tumor T-cell response [59]. Therefore, several reports point at the possibility to combine OVs, well-known inducers of ER stress, to drugs that are suboptimal inducers of ICD in order to improve the anti-tumor immune response.

### 4.2. OV Activity on Endothelial Cells and Cancer-Associated Fibroblasts

The indirect effects of OVs on cancer cells are not only mediated by immune cell conditioning, but are also due to effects on other cellular elements such as cancer-associated fibroblasts (CAFs) and endothelial cells. CAFs sustain tumor growth, angiogenesis, epithelial-to-mesenchymal transition (EMT), and promote tumor resistance to chemotherapeutic drugs [60,61].

Transforming growth factor-beta1 (TGF beta-1) is a key mediator in reshaping the phenotype of fibroblasts toward the CAFs phenotype, though TGF-beta suppresses IFN signaling [33]. This makes CAFs more susceptible to OV infection in comparison to normal fibroblasts because of reduced type I IFN. The susceptibility of cancer cells to OV infection has been demonstrated to increase when co-cultured with CAFs. Ilkow CS and *coll.* demonstrated that FGF2 produced by CAFs enhanced OV replication in cancer cells and reduced the anti-viral proteins produced by cancer cells in culture. Similarly, intra-tumor FGF2 or co-injection of CAFs increased OV replication in several xenograft models. Thus, the cross-talk between cancer cells and CAFs in the TME is crucial to determine OV sensitivity [62].

OVs exert an anti-angiogenic effect by acting on endothelial cells in TME [40]. Indeed, OVs target tumor-associated endothelial cells leading to a rapid destruction of the tumor vasculature and loss of perfusion within the tumor. It has been reported that vascular endothelial growth factor (VEGF) secretion by cancer cells makes endothelial cells susceptible to OV infection by reducing the anti-viral response. The cross talk between VEGF-driven pathways and interferon further inhibits the anti-viral response of endothelial cells [63]. Other mechanisms mediating disruption of tumor blood flow by OVs have been described, such as viral replication in tumor-associated endothelial cells [64]. OVs have been also reported to decrease the secretion of pro-angiogenic factors such as VEGF or IL-8 [65]. These effects can be exploited for synergistic combination with different anti-tumor agents [11].

## 5. OV Activity in TC Models

Several studies reported the efficacy of OV in TC experimental models either as single agents or in combination with anti-neoplastic drugs [66], main preclinical data are summarized in Table 1 and Table 2.

### 5.1. Oncolytic Ads Effects

Oncolytic Ads are the most studied OVs in ATC models. Ads can be easily produced and are suitable for manipulation to enhance their oncolytic activity. To date, a number of genetically modified oncolytic Ads have been developed and proved to be highly efficient.

*dl*1520, the first OV used against TC cell lines and in ATC xenografts, showed efficacy as a single agent [67]. However, in cell cultures, the combination with paclitaxel or doxorubicin induced cell death more efficiently than the single treatments. Indeed, its combination with ionizing radiation (IR) delayed the growth of ATC xenograft [68]. *dl*1520 has been also used in combination with lovastatin, a cholesterol-lowering drug that inhibits HMG-CoA reductase and consequently farnesylation and geranylgeranylation causing inactivation of Ras and Rho [69]. Lovastatin used alone exerts anti-proliferative effects in rat thyroid cells and induces apoptosis in human thyroid cells [69,70]. Moreover, Rho B is a relevant molecular target in ATC therapy [71]. The combination with *dl*1520 improved viral replication and cytotoxicity, enhancing ATC cell death [72]. This study is one of the first report showing that drugs inhibiting p21 RAS activity cooperate with OVs. Although several experimental studies supported the efficacy of *dl*1520 in ATC cells, these effects were not confirmed by clinical studies, thus novel OVs were generated [73,74] and tested in ATC models.

*dl*922-947 is a second generation Ads presenting a 24-bp deletion in the *E1A-conserved region 2* (CR2) [75] that is essential to bind and inactivate the pRb tumor suppressor protein. This mutation renders the virus unable to trigger S-phase entry in normal cells, whereas it can replicate only in cells with an aberrant G1-S checkpoint, a defect detected in the most of human cancers including ATC e PDTC [76]. In human cancer cells and also in ATC cells, *dl*922-947 presents a superior cell killing with respect to *dl*1520 [73,77,78,79,80,81]. In ATC experimental models *dl*922-947 has been extensively studied as a single agent [73] and also in combination with other drugs [73,82,83,84]. In particular, it was combined with bevacizumab, a humanized anti-VEGF antibody, to improve viral distribution and diffusion that is usually compromised by the abnormal tumor vascular structure and by the increased interstitial tumor pressure [85]. In ATC xenografts, the treatment with bevacizumab followed by virus administration normalized the vascular structure decreasing interstitial pressure, thus favoring a more efficient viral distribution within the tumor [73,86].

The effect of *dl*922-947 in ATC cells is usually associated with increase of DNA content (>4N) and accumulation in G2/M phase [84], likely because of abrogation of multiple cell cycle checkpoints by viral proteins. Thus, Libertini et al. hypothesized that the pharmacological block of the mitotic checkpoints could enhance *dl*922-947 efficacy. Considering that the mitotic kinase Aurora B is overexpressed in ATC cells and its inhibition reduces tumor growth [87], the combination with AZD1152, a specific Aurora B kinase inhibitor was investigated. AZD1152 induced G2/M phase accumulation, polyploidy, and cell death by mitotic catastrophe, its combination with *dl*922-947 showed additive or synergistic effects and induced a significant block of tumor growth [82].

To hinder viral infection, infected cells process viral DNA as damaged genetic material through the DNA damage response (DDR). To counteract DDR effects, viruses attempt to degrade or mislocalize key players of DDR [88,89,90]. In ATC cells, Passaro et al. demonstrated that *dl*922-947 triggers an inefficient DDR, since it induced accumulation of ɣH2AX, a marker of DNA damage [83].

The use of IR known to induce DNA damage was explored in association with *dl*922-947, when irradiation was administered prior to viral infection a synergistic tumor cell killing was observed [83]. *dl*922-947 was also used in association with KU55933, a specific-inhibitor of ATM kinase, this combination inhibited DDR enhancing the effects of *dl*922-947. The same group studied the activity of *dl*922-947 against other DNA repair pathways. In particular, poly (ADP-ribose) polymerase 1 (PARP-1) is a key mediator in base excision repair (BER) pathway and is also involved in other DNA damage repair processes, including the repair of DNA double strand breaks [91,92]. *dl*922-947 infection induces PARP-1 activation in ATC cells. Passaro et al. used Olaparib, a specific PARP inhibitor, in association with *dl*922-947; the combined effect greatly enhanced DNA damage accumulation and cell death in ATC cells [84]. These reports suggest that the exploitation of *dl*922-947 effects on DNA repair pathways might be of relevance to develop more effective therapeutic strategies against ATC.

Nonetheless, the impact of *dl*922-947 as single agent on the pro-tumorigenic ATC microenvironment was also evaluated. *dl*922-947 significantly reduced the production of inflammatory or pro-tumor cytokines and chemokines like IL-8/CXCL8 and MCP-1/CCL2 by infected ATC cells. Indeed, a reduced NF-κB p65 binding to IL-8 and CCL2 promoters in ATC cells infected with *dl*922-947 was demonstrated. The decrease of IL-8 secretion impaired the cell motility in vitro and tumor angiogenic response in vitro and in vivo. The decrease of the monocyte-attracting chemokine MCP-1/CCL2 reduced monocyte chemotaxis in vitro and tumor macrophage density in vivo. In ATC xenografts, *dl*922-947 induced the switch from M2 tumor-promoting macrophages toward a pro-inflammatory M1 phenotype. A high viral dose elicited tumor eradication in 40% of the mice after 2 weeks of treatment and the animals remained tumor-free until the end of the experiment. However, these effects were observed in immunocompromised animals, allowing only the evaluation of the innate response stimulated by the viral infection [65]. The confirmation in immune competent animals or in a more sophisticated immune competent model such as the humanized model, bearing the human immune system, could be useful. Nevertheless, these data clearly show that *dl*922-947 exerts an anti-tumor innate response and its direct effect on ATC cells is sufficient to modulate the TME.

Overall, the data obtained with *dl*922-947 sustain the possibility to use this virus or a more advanced version in ATC/PDTC patients.

Other Ads have been tested in ATC models. A fraction of undifferentiated TC presents mutations in beta-catenin gene [1]. This feature has been exploited to develop “HILMI,” an oncolytic Ads replicating in cells with an active Wnt/beta-catenin. In this virus a TCF response element drives the expression of adenoviral genes E1A and E1B, allowing viral replication in the fraction of ATC and PDTC cells with active Wnt/beta-catenin pathway. HILMI efficiently killed TC cells and prolonged the survival of mice with ATC xenografts [93].

In the oncolytic Ads ONYX-411, *E2F* promoter controls the expression of *E1A* and *E4* genes. This conditionally replicative oncolytic Ads was tested in TC experimental models [94]; however, no further studies were performed.

An adenoviral mutant (*dl*309) lacking *E3* region genes was tested in human K1 cells derived from PTC [95]. High levels of apoptosis were observed correlating to the expression of the viral *E1A* gene. *dl*309 virus has not been tested in cells derived from aggressive ATC/PDTC. *E3* gene products have a role in preventing innate immunity; however, the efficacy of *dl*309 to stimulate an immune response against cancer cells was not evaluated.

### 5.2. Other OV Effects

Multiple OVs other than adenoviral mutants have been tested as potential therapeutic agents in preclinical setting of ATC. Oncolytic vaccinia viruses (VACV) derived from vaccinia strains and related to cowpox virus have demonstrated efficacy both in preclinical and clinical studies [96]. Poxviruses used in other types of cancers demonstrated impressive partial and complete responses in a subset of patients. Mundi et al. used a combination of myxoma virus (MYXV) and tanapox virus (TPV), delivered either simultaneously or sequentially. MYXV, a member of *Leporipoxviridae* is a rabbit-specific poxvirus, its oncolytic potential is favored by the intracellular tumor environment. The oncolysis mechanism of MYXV includes phosphorylation of AKT and loss of synergistic effects of the tumor necrosis factor (TNF) and IFN responses in the neoplastic cells [97,98]. TPV, exhibiting a primate-specific tropism, represents an attractive candidate for oncolytic virotherapy. Its administration to humans induces only mild febrile illness, partially because TPV infection is usually limited to peripheral areas of the body. Indeed, except for endemic areas, person-to-person TPV transmission has not been observed. Overall, VACV showed more efficacy with respect to MYXV and TPV in controlling the viability and inducing death of ATC cells, thus this study confirmed the potential of VACV as a therapeutic agent against ATC/PDTC [99].

GLV-1h68 is a replication-competent VACV bearing mutations in three loci: *F14.5L, J2R* (encoding for thymidine kinase), and *A56R* (encoding for hemagglutinin). These mutations confer tumor selectivity to the virus and reduce its virulence in normal tissue. This virus also carries marker genes (*ruc-gfp*) that allow imaging of tumor-specific entry and replication [100]. GLV-1h68 showed significant inhibition of tumor growth after a single administration in ATC cells and tumor xenografts, [101,102]. A variant of GLV-1h68 carrying *hNIS* symporter gene was produced showing re-expression of *hNIS* in ATC cells [103].

The attenuated vaccine strain of measles virus, MVEdm, has been used in TC models. MVEdm has a specific tropism for CD46 receptor that is highly expressed on cancer cells, including TC [104,105,106]. Furthermore, to favor TC cell susceptibility to radioiodine therapy, the virus was modified to include the *NIS* symporter gene (MV-NIS). *NIS* expression both in vitro and in vivo was confirmed by radioactive iodine uptake along with single-photon emission computed tomography (SPECT) imaging of xenografts. However, ^131^I administration in MV-NIS-treated animals did not result in a significant enhancement of the effect, further studies on different ATC cells and with different treatment schedules are required [107].

NV1023 is a replication-competent HSV-1 attenuated by a 15 kb deletion in the inverted repeat region that extends from the 3′ end of UL55 to the promoter for ICP4. This virus was developed from NV1020, a non-selected clonal derivative of R7020, an HSV-1 vector initially designed as an HSV-1/2 vaccine candidate. NV1023 also contains the *Escherichia coli* β-galactosidase gene (*LacZ*) that serves as a marker of infection [108,109,110,111,112]. This virus showed cytotoxicity in one papillary, one medullary, and four anaplastic carcinoma cell lines. NV1023 oncolytic activity was confirmed in a xenografts model resulting in significant tumor volume reduction. Interestingly, a single administration of NV1023 in animals injected with papillary carcinoma cells elicited complete tumor resolution, in animals injected with anaplastic carcinoma cell lines significantly decreased the tumor volume however, the benefit of multiple dosing on tumor volume was not apparent [113]. In another study, a significant variation in the ability of NV1023 to enter TC cells was demonstrated evaluating the interaction between HSV-1 envelope glycoprotein D (gD) with one of three receptors (nectin-1, herpes virus entry mediator (HVEM), 3-O-sulfated heparin sulfate) required for viral entry. The relevance of the receptor Nectin-1 as marker of thyroid cancer sensitivity to herpes oncolytic therapy was validated [114]. In combinatory studies, Lin and colleagues used NV1023 and G207, an attenuated replication competent mutant HSV-1 with paclitaxel and adriamycin. G207 harbors mutations in both copies of the *γ 1 34.5* gene and a *lacZ* insertion in *UL39* gene that encodes the large subunit of the viral ribonucleotide reductase [108,109,110,111]. The combination of G207 with paclitaxel elicited synergy, whereas the association of NV1023 with adriamycin showed antagonistic effects [115].

The herpes OV, G47Δ presents the deletion of *γ34.5* gene that favors tumor cell selective cytotoxicity and virus replication in cells lacking innate immune response. The insertion of *lacZ* in the *UL39* gene inhibits the expression of ICP6, the large subunit of viral ribonucleotide reductase, thus avoiding viral DNA replication in normal cells. Furthermore, the ICP47 deletion enhances MHC class I presentation that activates lymphocytes and reduces NK cytolysis of host cells [110,116]. G47Δ showed a greater replication capability and a higher anti-tumor activity than G207, indeed elicited significant efficacy against ATC xenografts [116].

The oncolytic activity of Newcastle disease Virus strain FMW (NDV/FMW) has been also documented in ATC using a recombinant NDV carrying the GFP reporter gene. The tumor selectivity of NDV involves defects in anti-viral signaling pathways, in the activation of RAS signaling and the expression of Rac1 protein [117]. A significant and dose-dependent decrease of ATC cell viability was observed along with apoptosis activation. The efficacy of the recombinant virus was confirmed in 3D spheroids and in vivo models [118].

The therapeutic efficacy of BioKnife, an oncolytic recombinant Sendai virus that elicits cytotoxicity dependently on urokinase-type plasminogen activator activity has also been determined. Sendai virus is a natural OV lacking pathogenic effects in humans, but causing respiratory disease in mice. The administration of BioKnife prolonged survival in an orthotopic mouse model (BALB/c nu/nu mice) of ATC following intra-tumor injections [119], this finding also confirms the feasibility of the local treatments.

### 5.3. OV Effects in TC Patients

Clinical trials based on OVs in the treatment of ATC/PDTC have not been performed so far, likely because of the small number of patients and the short survival time. Nevertheless, in a Phase II clinical trial investigating the effects of a reovirus (REOLYSIN^®^) in combination with low-dose radiation in patients with advanced cancers, a single patient with TC was enrolled and displayed a partial response to the treatment (Intratumoral Administration of REOLYSIN^®^ in Combination with Low-Dose Radiation for Patients with Advanced Malignancies–Trial number REO 008).

Preclinical studies using VB-111, a non-replicating antiangiogenic Ads [120], supported its application in clinical trials. VB-111 is engineered to express Fas-c transgene in endothelial cells and is active only against tumor vasculature. In in vitro studies, infection of HUVEC cells induced massive apoptosis, whereas in animal models reduction of tumor mass and inhibition of metastasis was observed. In a phase I clinical trial, VB-111 proved to be safe and well-tolerated; with fever and chills as side effects. Transgene expression was detected in an aspirate from a subcutaneous metastasis but not in blood after treatment. In this trial, one patient with PTC had a partial response.

In a phase II clinical trial (ClinicalTrials.gov Identifier: NCT01229865, VB-111) in patients with advanced differentiated TC, 44% of patients experienced tumor regression. VB-111 is devoid of replicative activity, therefore this agent cannot be included among OVs. Overall, data from clinical studies support the use of viruses for the treatments of ATC and PDTC.

## 6. Future Treatments Based on OVs

Although OVs are proving to be effective as anti-cancer therapeutics, their efficacy as single agents is limited and combinations have been tested to enhance their antineoplastic effect.

The combinatorial treatments include the association of OVs with radiotherapy, chemotherapeutic drugs, and novel agents, including immune-checkpoint inhibitors (we refer to the following reviews for a better description of these studies) [121,122].

### 6.1. OVs and Chemotherapeutic Drugs

The combination of OVs with chemotherapeutics is an appealing strategy to increase anti-tumor potency. Different classes of chemotherapeutic agents have been tested in combination with OVs, showing in most cases additive or synergic effects both in preclinical and in clinical models [66]. With the exception of few studies, the mechanism at the base of synergy and increased cell killing was not analyzed.

It is emerging that chemotherapeutic drugs can elicit sub-optimal ICD because they fail to activate crucial DAMPs. In cisplatin-treated tumors although a release of ATP and HMGB1 was observed, the drug failed to induce cell surface CRT exposure [123]. Notably, OVs can induce the expression of broad arrays of DAMPs likely relevant for ICD induction.

Chemotherapeutic drugs (i.e., doxorubicin, paclitaxel) used against ATC have been associated with *dl*1520, the combinations showed to be more effective with respect to the single agents. In these studies, the capability of the combined treatment to potentiate ICD was not assessed [67]. The effects of 5-FU, gemcitabine, cisplatin, or low dose paclitaxel combined with *dl*922-947 have been tested in experimental models [65,77,124], but not assessed in ATC cells. The use of OVs with ICD inducers represents an important and promising future research direction, and it could be useful to identify ideal combinations in ATC setting. Moreover, the combination of ICD-inducing agents and OVs (as reported in pf 4.1) can minimize tumor escape variants at the base of treatment resistance.

### 6.2. OVs and Immunotherapeutic Agents/Treatments

Shaping an efficient immune response against tumors likely represents the most innovative therapeutic option available so far. Cancer immunotherapy is a clinically validated treatment for many solid tumors. Immunotherapy includes cancer vaccines, adoptive transfer of activated T or NK cells, the use of chimeric antigen receptor (CAR) T-cells, and blocking antibodies, the so called immune checkpoint inhibitors (ICI, i.e., anti-CTLA4 and anti-PD-1/PD-L1 antibodies) [125]. Immune checkpoint molecules are used by the immune system to maintain homeostasis, these signals are upregulated in tumors to escape the immune responses [126]. ICI are now approved for clinical use [127] and show efficacy in lesions with an existing anti-tumor immune response, whereas in tumors with minimal immune cells infiltration are less effective. Evidences suggest the use of ICI in combination with other agents capable of stimulating a robust immune response against neoplastic cells [128].

The analysis of the immunological features of ATC suggests that these tumors can take advantage of the immunotherapeutic option. Recently, transcriptomic and genomic data obtained by Cancer Genome Atlas (TCGA) provided a classification of the cancer stromal compartment including the immune component of solid tumor microenvironment. According to their “immunoscore,” tumors were classified into six types [129]. The immunoscore takes into account the tumors antigen burden, the phenotype, and degree of immune cell infiltration, the presence of soluble inhibitory mediators (i.e., TGF-beta, IL-10, VEGF), cell membrane inhibitory receptors (immune checkpoints) or immunosuppressive cells (i.e., myeloid derived suppressor cells, Treg). The TCGA analysis allowed the classification of PTCs as “inflammatory” tumors because of the recognition of an important immune infiltrate [129] despite their well-known low mutational burden. Furthermore, an immune expression profiling of TC confirmed that mostly ATC and PTC show a microenvironment infiltrated by macrophages and CD8^+^ T-cells with a functionally exhausted appearance [130]. These reports support the possible classification of the ATC as hot and immunosuppressed tumor and encourage the use of OVs as potential therapeutic option.

Several studies analyzed tumor-infiltrating immune/inflammatory cells in the aggressive forms of TC, and these data supported their role in mounting an anti-tumor response [131,132].

The transcriptomic analysis of TC samples revealed an up-regulation of inhibitory immune checkpoints (PD-1, PD-L1, and PD-L2) [125]. These datas were confirmed by other studies showing that (i) high PD-L1 levels in PTC were correlated with tumor-associated macrophages (TAM) and lymphocytic infiltrate [133,134]; (ii) the expression of PD-L1 was significantly associated with increased tumor size and multifocality of PDTC; (iii) PD-1^+^ T-lymphocytes were associated with cancer lymph-nodal invasion and recurrent disease [135].

Moreover, ATC cells express high levels of the enzyme indoleamine 2,3-dioxygenase 1 (IDO1) that catalyzes the conversion of the amino acid tryptophan to the immunosuppressive molecule kynurenines, thus causing halted growth of T cells [136].

The evidence of increased immune checkpoint expression and the existence of an inefficient immune infiltrate points to a promising role of immunotherapy. To date, few case reports presented the results of the effects of ICI in TC patients: pembrolizumab (anti-PD-1) has been used as a neoadjuvant drug allowing the complete surgical resection; in ATC patients, pembrolizumab was also used as salvage therapy added to kinase inhibitors [137,138]. More extensively, the utility of ICI in mono- or combination-therapy for advanced forms of TC are under investigation in several “basket clinical trials.” These trials are in phase I or II testing pembrolizumab, nivolumab (anti-PD-1), or atezolizumab (anti-PD-L1) alone or in combination with MKIs for advanced and/or metastatic TC (NCT02054806, NCT03246958 II, NCT02501096 and NCT02973997, NCT03181100, NCT01988896) [132].

OVs exert potent immune-stimulatory effects [139] and the lysis of cancer cells induces the release of antigens stimulating the anti-tumor immune response, thus turning “cold” immunosuppressive tumors to “hot” inflamed tumors. Indeed, OVs treatment favors effector T cell recruitment into the TME, and immune checkpoint blockade, via removal of inhibitory signals, and may sustain the potency of tumor infiltrating lymphocytes (TIL), TAM with an inflammatory M1 phenotype and NK cells. Reports strongly support the rationale for the use of OVs and ICI as therapeutic associations to stimulate the immune system against the tumor [125,140]. The efficacy of this strategy was confirmed in several studies and in different tumor types. Since the potential immune-stimulatory activity of *dl*922-947 is known in ATC models, a combination treatment with ICI and *dl*922-947 could be envisaged.

Another immunotherapeutic option is represented by the adoptive T-cell therapy. This approach has been successful for hematologic malignancies, yielding only modest results in solid tumors. In particular, preclinical results obtained in animal models, pointed at the possibility to consider CAR-T cell therapy targeting intercellular adhesion molecule 1 (ICAM-1) also for advanced human TC [141]. The association with OVs augmented the anti-tumor activity of adoptively transferred T-cells [140,142,143] in animal models increasing the survival of treated animals [144]. These studies proved that combining oncolytic virotherapy with adoptive T-cell immunotherapy is possible to control tumor growth.

Bispecific T cell engagers (BiTEs) consist of a CD3-scFv linked to another scFv specific for an antigen expressed on the surface of tumor cells. By utilizing BiTEs, T-cells infiltrating the tumor are re-directed against specific antigens expressed on cancer cells. OVs have been used to express BiTE molecules, providing a retargeting T-cell effect together with the virotherapeutic activity [142].

The development of novel treatments for melanoma can be considered as a paradigm for new anti-neoplastic approaches in different lesions, in particular TC as it shares several genetic lesions with melanoma cells. Bommareddy et al. combined trametinib, a selective MEK inhibitor (MEKi) and T-VEC, in melanoma experimental model observing a significant increase in cytotoxic activity independently of BRAF or NRAS mutation status. The combined treatment was associated with an increase in T-VEC replication. The TME showed an influx of proliferating CD8^+^ T cells expressing interferon-γ and Granzyme B. Authors observed an upregulation of PD-1 and PD-L1 gene expression in treated mice, suggesting that the therapeutic combination could benefit the PD-1/PD-L1 blockade. The triple combination with T-VEC/MEKi/αPD-1 was tested in an immune-competent model, showing nearly 80% of survival [145].

BRAF and RAS mutations represent early mutational events in ATC, and are present in 25% and 28% of cases, respectively [6]. BRAF inhibitors, or alternatively inhibitors active on the BRAF-MEK-MAPK signaling cascade, have been already used in ATC patients, and the combination with ICI has been already envisaged in a patient with unresectable ATC. Immunotherapy with pembrolizumab was combined with BRAF inhibitors enabling a complete surgical resection, as already above mentioned [137]. The triple combination MKIs, OVs, and ICI could be explored in ATC models to confirm the efficacy of this approach.

## 7. Conclusions

ATC and PDTC lack of effective therapies and novel treatments are required. Multimodality treatments have not increased the survival rate and the use of MKIs targeting the signaling pathways activated in TC have limited benefits.

OVs are now a clinical reality and have proved efficacy and safety. T-VEC is approved for the treatment of melanoma and its use has been already tested in other lesions in association with immunotherapy or other treatments [146].

The use of OVs for the treatments of ATC-PDTC has been hypothesized already in 2002, however, so far only preclinical studies have been performed in ATC/PDTC models and showed their efficacy against ATC cells and tumor xenografts. Indeed, the clinical administration of OVs in ATC/PDTC patients is limited by the short survival time. The compassionate treatment with OVs of ATC patients in advanced and untreatable stages could be envisaged in order to obtain clinical data and assess the capability of the viruses to stimulate an immune response.

Among the OVs tested in ATC/PDTC experimental models, the adenoviral mutant *dl*922-947 has been the most studied and its potency demonstrated either as single agent or in combination with other drugs and with IR. *dl*922-947 modulates the TME activating a robust innate immune response against ATC xenografts. These data confirm this virus as an attractive candidate for clinical studies. *dl*922-947 has already been evaluated in association with targeted therapeutics (i.e., Aurora B inhibitor) against ATC [82], although more combinations could be envisaged and might prove to be effective. Potentially active associations able to enhance the activity of OVs against TC cells might involve drugs endowed with activity against TC in preclinical and/or clinical studies like tyrosine kinase inhibitors (TKI) targeting multiple molecules or mTOR inhibitors.

Although data indicate that OVs and in particular *dl*922-947 could be beneficial for ATC/PDTC treatment, further studies could be useful to better address and develop therapeutic strategies based on OVs. These options of treatment might include the evaluation of OVs armed with immune-stimulatory genes (e.g., IL-12, GM-CSF, IL-2, IL-15, IFN-beta, IFN-gamma, TNF-alpha, CCL5, CCL2, CCL19, CXCL11) or the association with immunotherapeutic agents (e.g., ICI or co-stimulatory ligands) [27]. These combinations already have proved efficacy in other lesions (i.e., advanced melanoma), and could be effective in ATC/PDTC setting.

In conclusion, numerous studies report the efficacy of OVs in the TC preclinical scenario, thus supporting its clinical use in ATC patients in the near future.

## Figures and Tables

**Table 1:** DNA OVs.

Family and Type Species	Name	Viral Genetic Modification	Exploited Cancer Pathway	Combination with Other Therapeutics	Refs	Models
Adenoviridae, Human Adenovirus C	*dl*1520 (ONYX-015)	E1B-55kDa deletion	p53 deficiency	Doxorubicin, Paclitaxel	66	in vitro and mouse models
				Ionizing Radiation (IR)	68	
				Lovastatin	72	
	*dl*922-947	E1A-CR2 deletion	pRb deficiency	Bevacizumab	73	in vitro and mouse models
				AZD1152	82	
				IR	83	
				Olaparib	84	
	HILMI	TCF/β-catenin responsive promoter insertion	Wnt/*β*-catenin hyper-activation	NO	93	in vitro and mouse models
	ONYX-411	E2F promoter insertion controls E1A and E4 gene expression E1A-CR2 deletion	E2F hyper-expression pRb deficiency	NO	94	in vitro and mouse models
	*dl*309	E3 10.4K, 14.5 K, 14.7 K deletion E3 6.7K mutation		NO	95	in vitro
	VB-111 (non-replicative)	E1 deletion and insertion of Fas-c gene under a modified PPE-1 promoter		NO	120	mouse models
Poxviridae, Vaccinia Virus	VACV	EGFP insertion	AKT hyperactivation	NO	99	in vitro
	GLV-1h68	Insertion of RUC-GFP, β -galactosidase, β-glucuronidase into F14.5L, J2R and A56R loci of the viral genome, respectively	AKT hyperactivation	NO	101-103	in vitro and mouse models
Poxviridae, Tanapox Virus Myxoma Virus	TANV MYXV	EGFP insertion	AKT hyperactivation	NO	99	in vitro
Herpesviridae, Human herpesvirus 1	NV1023	HSV2 genes (US2-2 and US2-5) insertion in the UL/S junction. ICP10, ICP4, γ34.5 gene deletion. *lacZ* gene insertion into US10-12 locus.	Defective PKR signaling	NO	108	mouse models
	G207	γ34.5 deletion UL39 inactivation by insertion of *lacZ* gene	Defective PKR signaling	Paclitaxel and Adriamycin	109	in vitro and mouse models
	G47Δ	γ34.5, ICP47 and US11 promoter deletions UL39 inactivation by insertion of *lacZ* gene	Defective PKR signaling	NO	110	in vitro and mouse models

**Table 2:** RNA OVs.

Family and Type Species	Name	Viral Genetic Modification	Exploited Cancer Pathway	Combination with other therapeutics	Refs	Model
Paramyxoviridae (*Negative ssRNA*), Measles Virus (MV)	MV-Edm	Live attenuated Edmonston B strain	CD46 expression on cell surface		104-106	in vitro and mouse models
	MV-NIS	Human NIS insertion	CD46 expression on cell surface	Radio iodine therapy (^131^I)	107	in vitro and mouse models
Paramyxovirus *(ssRNA)*, Newcastle disease virus (NDV)	NDV/FMW	Unmodified oncolytic NDV, FMW strain	RAS hyperactivation	NO	117	in vitro, mouse models and clinical studies
Paramyxovirus *(ssRNA)*, Sendai virus	BioKnife	M gene deletion and insertion of GFP gene Insertion of uPA cleavage site in F gene	uPA activity	NO	119	in vitro and mouse models
Reoviridae *(dsRNA)*, Reovirus	REOLYSIN	Unmodified oncolytic Reovirus	Defective (PKR) signaling RAS activation Defective antiviral response Cellular stress	Low dose IR (PHASE II study for advanced cancer, partial response in one patient with thyroid carcinoma)	54	in vitro and mouse models

## Abbreviations

APC: antigen presenting cells; ATC, anaplastic thyroid carcinoma; BiTEs, bispecific T cell engagers; CAFs, cancer-associated fibroblasts; CAR-T, chimeric antigen receptor T-cells; CRT, calreticulin; DAMPs, danger associated molecular patterns; DCs, dendritic cells; DDR, DNA damage response; ECHO-7, non-pathogenic enteric cytopathic human orphan type 7; EMT, epithelial-to-mesenchymal transition; FTC, follicular thyroid carcinoma; HSV-1, herpes simplex virus 1; hTert-Ad, human telomerase reverse transcriptase promoter-regulated Ads; ICD, immunogenic cell death; IDO1, indoleamine 2,3-dioxygenase 1; IFNs, interferons; IR, ionizing radiation; MEKi, MEK inhibitor; MKIs, multiple kinase inhibitors; MYXV, oncolytic myxoma virus; NDV/FMW, Newcastle disease Virus NDV strain FMW; NK, natural killer cells; OV, oncolytic viruses; PARP-1, poly (ADP-ribose) polymerase 1; PARP-2, poly (ADP-ribose) polymerase 2; PDTC, poorly differentiated thyroid carcinoma; PKR, protein kinase R; PPE-1, preproendothelin-1; PRRs, pathogen recognition receptors; PTC, papillary thyroid carcinoma; SPECT, single-photon emission computed tomography; TC, thyroid carcinoma; TGF beta-1, transforming growth factor-beta1; TK, tyrosine kinase; TKI, tyrosine kinase inhibitors; TLR, toll-like receptor; TME, tumor microenvironment; T-VEC, talimogene laherparepvec; VEGF, vascular endothelial growth factor.
